# Current status and challenges of colorectal cancer screening program in Guangzhou, China: a population-based study

**DOI:** 10.1093/gastro/goag011

**Published:** 2026-05-19

**Authors:** Dan Zhao, Boheng Liang, Yingru Liang, Suixiang Wang, Huan Xu, Jun Yuan

**Affiliations:** School of Public Health, Southern Medical University, No. 1023–1063, Shatai South Road, Baiyun District, Guangzhou, Guangdong, P. R. China; Guangzhou Center for Disease Control and Prevention, No.1 Qide Road, Jiahe, Baiyun District, Guangzhou, Guangdong, P. R. China; Guangzhou Center for Disease Control and Prevention, No.1 Qide Road, Jiahe, Baiyun District, Guangzhou, Guangdong, P. R. China; Guangzhou Center for Disease Control and Prevention, No.1 Qide Road, Jiahe, Baiyun District, Guangzhou, Guangdong, P. R. China; Guangzhou Center for Disease Control and Prevention, No.1 Qide Road, Jiahe, Baiyun District, Guangzhou, Guangdong, P. R. China; Guangzhou Center for Disease Control and Prevention, No.1 Qide Road, Jiahe, Baiyun District, Guangzhou, Guangdong, P. R. China; School of Public Health, Southern Medical University, No. 1023–1063, Shatai South Road, Baiyun District, Guangzhou, Guangdong, P. R. China; Guangzhou Center for Disease Control and Prevention, No.1 Qide Road, Jiahe, Baiyun District, Guangzhou, Guangdong, P. R. China

**Keywords:** colorectal cancer, cancer screening, cross-sectional study

## Abstract

**Background:**

Colorectal cancer (CRC) remains a major public health burden. In 2015, a large-scale population-based CRC screening program was launched in Guangzhou for residents aged 45–74 years. This study assessed the program’s performance during 2015–2023 to inform future strategies.

**Methods:**

A cross-sectional analysis was conducted by using data from 575,843 first-time participants. High-risk individuals were identified through a risk assessment questionnaire combined with fecal immunochemical test (FIT), with positives referred for colonoscopy. Statistical analyses were weighted according to the 2020 Guangzhou census population. Passive follow-up was performed via the Guangzhou Cancer Registration System, enabling estimation of true positives and false negatives. Group differences were examined by using the *χ*^2^ test, and factors associated with participation and lesion detection were analyzed by using multivariate logistic regression models.

**Results:**

Of 575,843 participants, 83,590 (14.52%; weighted, 14.55%) were assessed as high-risk. Among them, 22,157 (26.51%; weighted, 28.17%) completed colonoscopy. Overall, 915 CRC cases were detected. Higher colonoscopy adherence was associated with higher education, family history of CRC, anorectal symptoms, and financial allowance. Lesions were found to be more common in older individuals, men, and those with positive FIT. More low-risk lesions and fewer early- and mid/late-stage CRCs were found during the second round of screening (2021–2023) than during the first round of screening (2015–2017). Passive follow-up identified 233 CRCs within 1 year among initially screening-negative participants.

**Conclusions:**

This study demonstrated the feasibility of a two-step CRC screening strategy in Guangzhou and its effectiveness in detecting early lesions. However, poor adherence to follow-up colonoscopy remains a key limitation. Targeted interventions—including health education, system-level support, and appropriate financial incentives—are essential to improve compliance and overall program effectiveness.

## Introduction

Colorectal cancer (CRC) is one of the most common cancers worldwide, with 1.93 million new cases in 2020. Notably, it is the third most prevalent cancer and the second leading cause of cancer mortality, following lung cancer [1]. Statistically, CRC ranks as the third most common cancer in men and the second most common cancer in women [2]. However, trends in CRC incidence and mortality vary significantly between developed and developing countries.

Overall, a decline trend or stabilization of CRC incidence has been observed in developed countries such as the European region, the United States, and Japan [3–5]. This trend may be attributed to the early establishment of nationwide screening programs and increased colonoscopy rates, alongside lifestyle and dietary changes [6]. In contrast, the burden of CRC continues to rise rapidly in developing regions, with morbidity and mortality increasing notably in Africa, Asia, and Latin America [3, 7]. In addition, CRC is increasingly diagnosed in younger populations under 50, with the incidence rising annually [8, 9].

As a country undergoing an epidemiological and socioeconomic transition, China reflects these changes in CRC incidence [10]. In 2020, China reported 555,000 new CRC cases and 286,000 deaths, ranking second in incidence and fifth in mortality globally [11]. In Guangzhou, a megacity in China, CRC incidence and mortality rates have risen significantly over the past decade: the crude incidence rate of CRC increased from 22.95 to 52.81 per 100,000, and the crude mortality rate from 4.33 to 24.89 per 100,000 [12].

In response to this growing burden, Guangzhou launched a large-scale population-based CRC screening program in 2015, targeting residents aged 45–74 years. The program employed a two-step screening approach—risk assessment by questionnaire and FIT, followed by colonoscopy for high-risk individuals. Over the past 9 years, this program has accumulated one of the largest CRC screening datasets in South China.

Therefore, we present a comprehensive evaluation of the screening program from 2015 to 2023. We assessed participation in initial screening, adherence to colonoscopy, lesion detection outcomes, and associated factors. Understanding which aspects of the program are practical and which require improvement is essential for informing future screening strategies. Importantly, this study provided a comprehensive evaluation of current screening strategies, identifying key challenges and offering evidence-based suggestions for their future optimization.

## Methods

### Target population

The Guangzhou Colorectal Cancer Screening Program is a government-led public health initiative coordinated by the Guangzhou Municipal Health Commission (Guangzhou, China). Annual screening targets were allocated to each administrative district based on population size and healthcare capacity. The program targeting residents aged 45–74 in Guangzhou included both household-registered and non-registered residents who have lived in the city for at least 6 months. Community Health Centers (CHCs) in each district identified eligible participants through household registration and residency records. With support from neighborhood committees, CHCs mobilized residents using posters, phone calls, and in-person communication. Online channels such as WeChat public accounts, WeChat Moments, and Douyin videos were also employed to expand outreach. Traditional media—including television, radio, and community bulletin boards—were used to promote participation further.

Although participants were not selected through probabilistic sampling, the structured recruitment method under district-level screening quotas enabled broad population coverage. Similar community-based mobilization strategies have been adopted in other large-scale Chinese screening initiatives, such as the Cancer Screening Program in Urban China (CanSPUC) [13] and the Shanghai CRC screening program [14].

This study analyzed data from individuals who underwent CRC screening between 2015 and 2023. All participants were thoroughly informed of the potential risks and benefits of CRC screening and provided written informed consent before participating. Individuals aged below 45 or above 74 were excluded, even if they volunteered. Inclusion criteria for this study were as follows: (i) Guangzhou household residents or non-household residents who have lived in the city for >6 months and (ii) participation in screening from January 2015 to December 2023. Exclusion criteria included (i) residents with severe cardiopulmonary, cerebral, or psychiatric diseases who cannot participate in colonoscopy screening, (ii) residents with reading, writing, or listening disabilities who cannot cooperate in completing questionnaire assessments or follow-up visits, or (iii) residents who currently have CRC or have been previously diagnosed with CRC. For this study, participants were further restricted to those who also met the following conditions: (i) first-time participation in CRC screening and (ii) no prior history of colonoscopy, regardless of whether it was performed within or outside the screening program. Notably, these additional criteria were applied to ensure a homogeneous study population and minimize potential bias related to prior screening or diagnostic procedures.

### Initial screening process

Following informed consent, high-risk individuals were identified through questionnaire-based interviews collecting data on risk factors (such as demographics, previous anorectal symptoms, and previous history of non-colon cancer), combined with two rounds of fecal occult blood test (FOBT) results. Positive judgment criteria for the questionnaire (Colorectal Cancer Quantitative Questionnaire for Screening Risk Factors [15]) assessment included (i) history of CRC in a first-degree relatives (FDRs), (ii) personal history of previous cancer (excluding colon cancer), (iii) personal history of colon polyps, and (iv) two or more of the following conditions: chronic diarrhea, chronic constipation, mucus or bloody stools, chronic appendicitis or history of appendectomy, chronic cholecystitis or history of cholecystectomy, and major traumatic or distressing events affecting mental health. A positive questionnaire assessment was determined by meeting any of these criteria listed above. The FOBT was an immunochemical test based on colloidal gold technology (immunochemical fecal occult blood test, iFOBT), also referred to as the fecal immunochemical test (FIT). There was a 1-week interval between the two FITs. If either test returned positive, the overall FIT was considered positive. Notably, participants with a positive result from either the questionnaire assessment or the FIT were defined as high-risk. Since some participants tested positive on both assessments, the final number of high-risk individuals was based on the deduplicated union of both groups.

This two-step strategy—combining a quantitative risk factor questionnaire with FIT—is consistent with the Chinese Expert Consensus on Early Diagnosis and Treatment of Colorectal Cancer, as well as national programs such as the CanSPUC. Similar approaches have been successfully applied in large-scale CRC screening initiatives in Zhejiang Province, including Jiaxing and Haining, demonstrating both high detection rates of advanced lesions and reductions in CRC mortality among screened populations [16, 17].

### Colonoscopy-based clinical screening

High-risk CRC participants were referred to designated hospitals for a total colonoscopy performed by an experienced gastroenterologist, who was an attending or higher-level physician with at least 5 years of endoscopy experience. The scope should reach the ileocecal region, with a withdrawal time of at least 6 minutes when conditions permit. Any abnormal findings were recorded, and biopsies were taken for subsequent pathological diagnosis. Patients with inadequate bowel preparation repeated the colonoscopy to meet clinical diagnostic criteria. For complex cases, the panel of experts from the Guangzhou Digestive Disease Center was consulted, and the results were forwarded to the respective doctors. Diseases detected by colonoscopy included CRC (early- and mid/late-stages), advanced adenomas, non-advanced adenomas, non-adenomatous polyps, and inflammatory diseases. Some diseases were defined below: early-stage cancer refers to CRC classified as T1–2N0M0; advanced adenomas are defined as adenomas >10 mm, or containing villous structure, or with high-grade intraepithelial neoplasia [15]. As a further clarification, unstaged CRC refers to CRC cases for which TNM staging information was not available in clinical or registry records.

### Quality control and data management

A comprehensive quality control framework was also implemented to ensure procedural consistency, data reliability, and participant safety throughout the 9-year program. Personnel from medical institutions and disease prevention agencies, including clinicians, endoscopists, laboratory technicians, and public health doctors, received systematic training in CRC screening protocols, specimen handling, database management, and quality assurance procedures. All CHCs adhered to standardized operating procedures issued by the Guangzhou Municipal Health Commission. Questionnaire completion followed strict survey protocols, with on-site review to minimize omissions and logical errors. Fecal sample collection was conducted according to established guidelines, with seasonal scheduling to avoid high-temperature periods. Colonoscopy equipment underwent regular maintenance and sterilization in compliance with clinical hygiene standards. Furthermore, contingency plans were established to manage unexpected situations and ensure participant safety throughout the screening process.

All screening data were entered into the Guangzhou Colorectal Cancer Screening Registration and Management System [18], a centralized electronic platform established in 2015. This system captured all relevant fields, including demographic information, risk assessment questionnaire results, FIT results, initial screening results, and colonoscopy examination results. Each participant was assigned a unique screening code, and identity verification was performed by using national ID card numbers during data entry. The system automatically checked for duplicate records, coding errors, and logical inconsistencies, providing real-time prompts to facilitate error correction. In addition, built-in reminder functions supported follow-up appointment scheduling, enabling timely contact with high-risk individuals and promoting colonoscopy adherence. Routine data audits were carried out by the Guangzhou Center for Disease Control and Prevention to identify and resolve discrepancies, thereby ensuring data consistency across districts and time periods. To account for temporal variations in participant characteristics and implementation practices, the variable “year of screening” was included in all multivariate models.

### Follow-up strategy and outcome ascertainment

Active follow-up was conducted for participants with positive screening results, who were required to complete a colonoscopy within 10 months. CHCs coordinated this follow-up process through structured steps: the first contact was made within 1 month of a positive initial screening result. If a colonoscopy was not completed, a second follow-up was conducted at 3 months. A third follow-up was performed within 6 months after the second contact if colonoscopy remained incomplete. Participants who failed to undergo colonoscopy after all three follow-up attempts were classified as non-adherent, and their outcomes were included in long-term surveillance. For screening-negative individuals, passive follow-up was performed by linking their unique ID card numbers with the Guangzhou Cancer Registry to obtain incident CRC diagnoses as of 30 June 2024. This registry linkage ensured comprehensive identification of CRC cases among screening-negative participants and represents an internationally recognized approach for long-term outcome monitoring in large-scale, population-based screening programs.

True positives (TP) were defined as screening-positive individuals who completed a colonoscopy within 10 months and were diagnosed with CRC. False positives (FP) referred to screening-positive individuals who completed a colonoscopy and were not diagnosed with CRC. False negatives (FN) were defined as screening-negative individuals who were diagnosed with CRC within 12 months after initial screening. True negatives (TN) included screening-negative individuals without a CRC diagnosis within the same period. Finally, CRC cases diagnosed >12 months after screening were analyzed separately as long-term outcomes.

### Statistical analysis

Data management and statistical analyses were performed by using Excel 2016, R (Version 4.3.3), and IBM SPSS 20.0. To address missing colonoscopy results from both the initial and diagnostic screening phases, records were retrieved via the Guangzhou Colorectal Cancer Screening Registration and Management System and the Guangzhou Cancer Registration and Management System. The Guangzhou Center for Disease Control and Prevention managed both. For other variables with missing values, multiple imputation was applied to ensure data completeness and analytical accuracy [19]. To enhance population representativeness, all analyses were conducted on data weighted by the age and gender distribution of the 2020 Guangzhou census (Table S1). Weighting was applied prior to statistical analyses to correct for discrepancies between the study sample and the general population structure.

Descriptive statistics were used to summarize the baseline characteristics. Weighted proportions of key outcomes, such as the composition ratio of initial screening participants, questionnaire positivity, FIT positivity, initial screening-positive rate, and colonoscopy adherence, were calculated across different subgroups. Differences in these rates between groups were assessed by using the *χ*^2^ test. A multiple logistic regression model was used to identify factors associated with colonoscopy adherence among individuals with positive initial screening results. Independent variables included in the final model were those found to be statistically significant in univariate *χ*^2^ tests and without evidence of multicollinearity (variance inflation factor, VIFs < 5; Table S2) [20]. Covariates included gender, age group, marital status, education level, occupation, previous anorectal symptoms, non-colon cancer, family history, FIT results, and year of enrollment. Adjusted odds ratios (ORs) and 95% confidence intervals (CIs) were reported. Additionally, we estimated the detection rate of each lesion type identified during colonoscopy. We also identified the number of colonoscopies required to detect 1 case per 10,000 initially screened-positive participants. Factors associated with lesion detection were assessed by using multinomial logistic regression, with results expressed as ORs and 95% CIs. Prior to multivariate analyses, multicollinearity diagnostics were performed for all covariates, confirming no significant multicollinearity (all VIFs < 5, Table S3). All statistical analyses were two-tailed, with a significance level of *α* (*α* = 0.05).

## Results

### Baseline characteristics of screening program participants from 2015 to 2023

**Figure 1:**
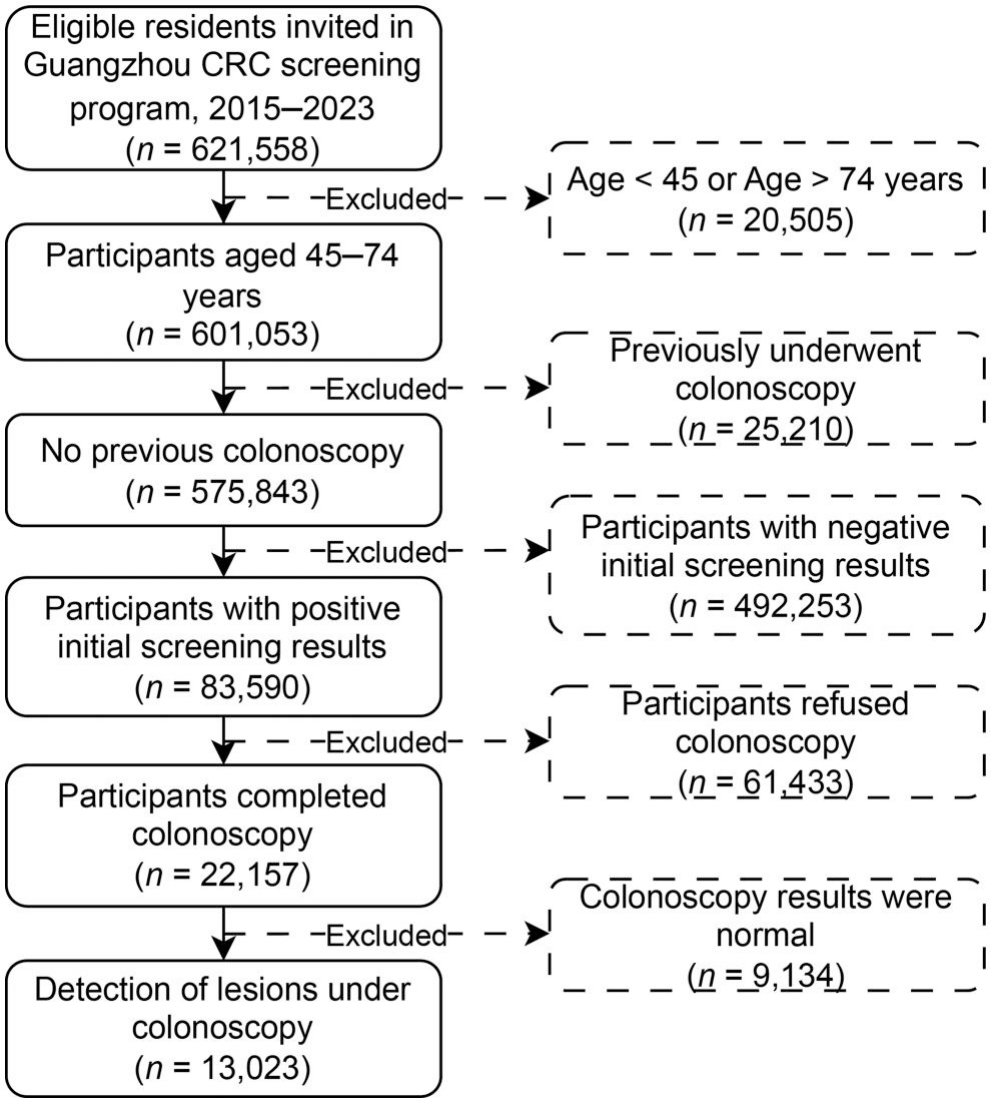
Colorectal cancer screening program participant recruitment flowchart.

As shown in Fig. 1, 621,558 eligible individuals were recruited into the Guangzhou Colorectal Cancer Screening Program between 2015 and 2023. After excluding those not in the 45–74 age range (*n *= 20,505) and those with a history of previous colonoscopy (*n *= 25,210), we included 575,843 participants undergoing initial screening for the final analyses. Among all these participants, 38.02% were men and 61.98% were women. The mean age was 61.47 years (SD = 7.13 years), and the majority (41.51%) were between 65 and 74 years old. Almost all participants (93.07%) were married, 89.43% had intermediate or lower levels of education, and the occupational distribution is mainly in other industries (36.99%).

To improve the representativeness of our findings, post-stratification weighting was applied based on the age and gender distribution of the Guangzhou population, according to the Seventh National Census. After weighting, the gender distribution became more balanced (48.29% were men and 51.71% were women). The age distribution also shifted, with most participants now in the 45–54 age group (51.16%), followed by 55–64 (31.12%) and 65–74 (17.72%). Other demographic variables, such as education level, marital status, and occupation, showed slight changes after weighting (Table 1).

**Table 1:** Demographic characteristics of the study population before and after weighting to the age-sex distribution of the general population in Guangzhou.

Characteristic	Unweighted		**Weighted** ^a^	
	Screened participants	Proportion (%)	Screened participants	Proportion (%)
**Gender**				
Men	218,946	38.02	158,083	48.29
Women	356,897	61.98	169,270	51.71
**Age groups**				
45–54	121,085	21.03	167,476	51.16
55–64	215,732	37.46	101,886	31.12
65–74	239,026	41.51	57,991	17.72
**Regions**				
Baiyun	82,498	14.33	47,647	14.56
Conghua	28,338	4.92	18,631	5.69
Panyu	63,711	11.06	36,517	11.16
Haizhu	83,256	14.46	42,741	13.06
Huadu	44,791	7.78	26,910	8.22
Huangpu	29,283	5.09	17,544	5.36
Liwan	57,100	9.92	29,978	9.16
Nansha	18,465	3.21	10,783	3.29
Tianhe	50,563	8.78	27,584	8.43
Yuexiu	68,346	11.87	37,841	11.56
Zengcheng	49,492	8.59	31,179	9.52
**Marital status**				
Married	535,955	93.07	309,675	94.60
Single/divorced/widowed	39,888	6.93	17,679	5.40
**Education**				
Low	211,850	36.79	96,844	29.58
Intermediate	303,103	52.64	185,335	56.62
High	60,890	10.15	45,174	13.80
**Occupation**				
Enterprise	133,427	23.17	76,620	23.41
Organizations/institutions	51,458	8.94	33,457	10.22
Agriculture	138,072	23.98	72,964	22.29
Freelance	39,894	6.93	34,746	10.61
Other	212,992	36.99	109,567	33.47
**Total**	**575,843**	100.00	**327,353**	100.00

a Weighted data were adjusted using post-stratification weights derived from the 2020 Guangzhou Census. Proportions are presented as percentages.

### Post-stratification weighted positivity rates by sociodemographic characteristics

After applying post-stratification weighting, the positivity rates for questionnaire-only and FIT-only screening were 9.26% and 6.77%, respectively. Among those who completed the initial screening, 14.55% tested positive. The positivity rate was higher in men than in women (15.16% vs 13.99%, *P *< 0.001). No significant differences were observed across age groups (*P *= 0.286). However, the positivity rate increased with higher education levels (*P *< 0.001, test of trend). Married participants had a lower positive rate compared to those who were single, divorced, or widowed (14.31% vs 18.81%, *P *< 0.001). Furthermore, statistically significant differences were also observed across regions (*P *< 0.001) and occupations (*P *< 0.001) (Table 2).

**Table 2:** Characteristics of participants with questionnaire positive, FIT-positive, and initial screening positive.

Characteristic	**Questionnaire positive only, *N* (%)** ^a^	***P* values** ^b^	**FIT-positive only, *N* (%)** ^a^	***P* values** ^b^	**Initial screening positive*, N* (%)** ^a^	***P* values** ^b^
**Gender**						
Men	15,029 (9.51)	<0.001	10,602 (7.33)	<0.001	23,961 (15.16)	<0.001
Women	15,283 (9.03)		9,788 (6.26)		23,682 (13.99)	
**Age groups**						
45–54	17,146 (10.24)	<0.001	8,608 (5.60)	<0.001	24,266 (14.49)	0.286
55–64	8,809 (8.65)		7,002 (7.46)		14,819 (14.54)	
65–74	4,357 (7.51)		4,779 (8.97)		8,558 (14.76)	
**Regions**						
Baiyun	3,091 (6.49)	<0.001	2,744 (6.31)	<0.001	5,568 (11.69)	<0.001
Conghua	768 (4.12)		1,243 (6.84)		1,930 (10.36)	
Panyu	2,896 (7.93)		1,667 (4.83)		4,373 (11.98)	
Haizhu	4,684 (10.96)		2,706 (6.97)		6,918 (16.19)	
Huadu	1,754 (6.52)		1,578 (5.97)		3,126 (11.61)	
Huangpu	1,491 (8.50)		997 (5.89)		2,372 (13.52)	
Liwan	2,659 (8.87)		2,081 (7.49)		4,441 (14.81)	
Nansha	873 (8.10)		548 (5.17)		1,330 (12.34)	
Tianhe	3,813 (13.82)		1,930 (7.92)		5,378 (19.50)	
Yuexiu	6,406 (16.93)		3,227 (10.98)		8,907 (23.54)	
Zengcheng	1,877 (6.02)		1,669 (5.48)		3,300 (10.58)	
**Marital status**						
Married	27,892 (9.01)	<0.001	19,231 (6.74)	0.005	44,317 (14.31)	<0.001
Single/divorced/widowed	2,419 (13.68)		1,159 (7.32)		3,326 (18.81)	
**Education**						
Low	5,054 (5.22)	<0.001	5,564 (6.14)	<0.001	10,015 (10.34)	<0.001
Intermediate	17,649 (9.52)		12,038 (7.08)		27,882 (15.04)	
High	7,609 (16.84)		2,789 (6.93)		9,745 (21.57)	
**Occupation**						
Enterprise	9,397 (12.26)	<0.001	5,269 (7.61)	<0.001	13,793 (18.00)	<0.001
Organizations/institutions	4,193 (12.53)		2,012 (6.73)		5,814 (17.38)	
Agriculture	3,414 (4.68)		4,021 (5.74)		7,014 (9.61)	
Freelance	3,714 (10.69)		1,925 (6.00)		5,303 (15.26)	
Other	9,594 (8.76)		7,163 (7.18)		15,718 (14.35)	
**Total**	**30,311 (9.26)**		**20,390 (6.77)**		**47,643 (14.55)**	

a The number of positive cases and the positive rate after weighting.

b The *P* values from *χ*^2^ analysis of different subgroups. FIT = fecal immunochemical test.

### Colonoscopy adherence rate and its influencing factors

Between 2015 and 2023, 22,157 individuals with positive initial screening results underwent colonoscopy, resulting in an adherence rate of 26.51% (22,157/83,590). After weighting by gender and age, 13,421 participants were estimated to have completed a colonoscopy, corresponding to a weighted adherence rate of 28.17%. Subsequent regression analyses were conducted by using the weighted dataset to represent the underlying population structure better.

**Figure 2:**
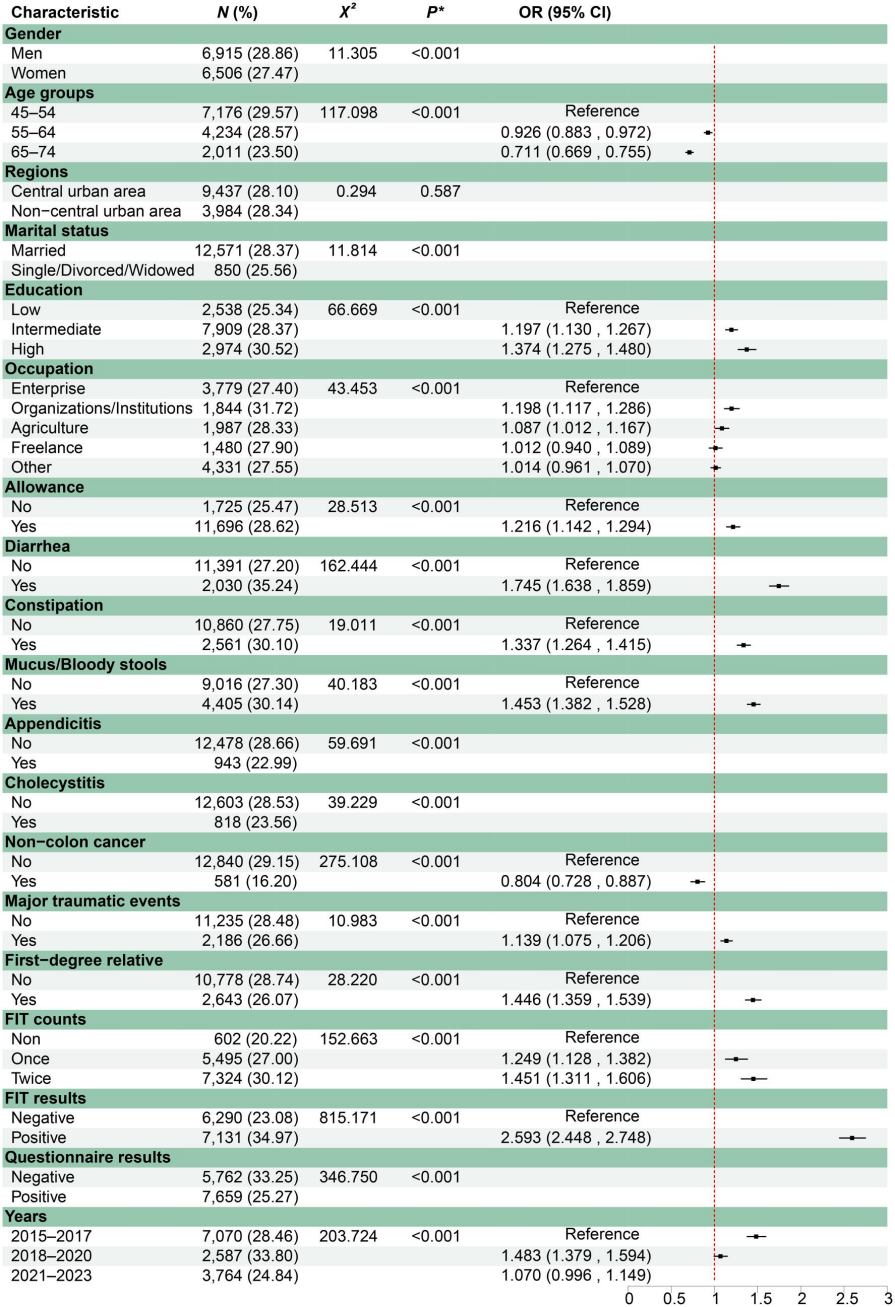
Factors associated with adherence rate in colonoscopy in the initial screening program. Analyses were weighted by sex and age; P* indicates *P* values from one-way χ^2^ test.

As presented in Fig. 2, the adherence rate decreased with the increase in age. Significantly lower odds were observed in participants aged 55–64 years (OR = 0.926, 95% CI: 0.883–0.972) and 65–74 years (OR = 0.711, 95% CI: 0.669–0.755) than in the 45–54 years reference group. High educational level was positively associated with colonoscopy adherence. Participants who received colonoscopy allowance also had a higher adherence rate than those who did not (OR = 1.216, 95% CI: 1.142–1.294). Individuals reporting anorectal symptoms exhibited higher adherence, including those with chronic diarrhea (OR = 1.745, 95% CI: 1.638–1.859), chronic constipation (OR = 1.337, 95% CI: 1.264–1.415), and mucus/bloody stools (OR = 1.453, 95% CI: 1.382–1.528), than individuals without the corresponding symptom, respectively. In contrast, participants with a history of non-colon cancers showed lower adherence than their counterparts with no such history (OR = 0.804, 95% CI: 0.728–0.887). Experiencing a major traumatic event (OR = 1.139, 95% CI: 1.075–1.206) and having a first-degree relative with CRC (OR = 1.446, 95% CI: 1.359–1.539) were both positively associated with colonoscopy adherence. Additionally, individuals who tested positive on FIT showed a higher adherence rate than those who were FIT negative (OR = 2.593, 95% CI: 2.448–2.748).

### Colon lesions detected by colonoscopy

Among the 22,157 participants who underwent colonoscopy, 13,023 lesions were identified. This yielded an overall lesion detection rate of 58.78%. Specifically, 6,201 (27.99%) non-adenomatous polyps, 3,729 (16.83%) non-advanced adenomas, and 2,178 (9.83%) advanced adenomas were detected. A total of 915 (4.13%) CRCs were detected, including 313 (1.41%) early-stage CRCs, 416 (1.88%) mid/late-stage CRCs, and 186 (0.84%) unstaged CRCs. Early-stage CRC accounted for 34.21% of all stages of CRC cases. Weighted analysis adjusted for the 2020 Guangzhou population structure showed similar detection patterns: 27.92% for non-adenomatous polyps, 16.58% for non-advanced adenomas, and 8.74% for advanced adenomas. The weighted detection rate of CRC was 3.04%, including 0.95% early-stage CRC, 1.41% mid/late-stage CRC, and 0.68% unstaged CRC.

To assess diagnostic efficiency, we calculated the number of colonoscopies that was required to detect each type of lesion based on weighted data. On average, 1 case of non-advanced adenoma was detected per 6 colonoscopies, 1 advanced adenoma per 11 procedures, and 1 CRC per 33 colonoscopies. Specifically, 105 colonoscopies were needed to detect 1 early-stage CRC, 71 for 1 mid/late-stage CRC, and 146 for 1 unstaged case. In terms of weighted yield, per 10,000 individuals with positive initial screening results, colonoscopy identified 787 non-adenomatous polyps, 468 non-advanced adenomas, 247 advanced adenomas, and 86 CRC cases, including 27 early-stage, 40 mid/late-stage, and 20 unstaged cancers (Table 3).

**Table 3:** Colon lesions detected by colonoscopy in a screening program.

Detection of disease	**Detected cases (%)** ^a^		**Yield per 10,000 initial screening positive participants** ^b^		**Colonoscopies to detect one lesion** ^c^	
	Unweighted	Weighted	Unweighted	Weighted	Unweighted	Weighted
Non-adenomatous polyps	6,201 (27.99)	3,747 (27.92)	742	787	4	4
Non-advanced adenomas	3,729 (16.83)	2,225 (16.58)	446	468	6	6
Advanced adenomas	2,178 (9.83)	1,173 (8.74)	261	247	12	11
CRC	915 (4.13)	408 (3.04)	109	86	28	33
Early-stage CRC	313 (1.41)	127 (0.95)	37	27	83	105
Mid/Late-stage CRC	416 (1.88)	189 (1.41)	50	40	64	71
Unstaged CRC	186 (0.84)	92 (0.68)	22	20	120	146

a Detected cases (%) = Number of detected cases/Total number of colonoscopies.

b Yield per 10,000 initial screening positive participants = 10,000 *completed colonoscopy rate* case detection rate.

c Colonoscopies to detect one lesion = 1/Detection rate of case.

### Factors associated with CRC detection in colonoscopy

**Figure 3:**
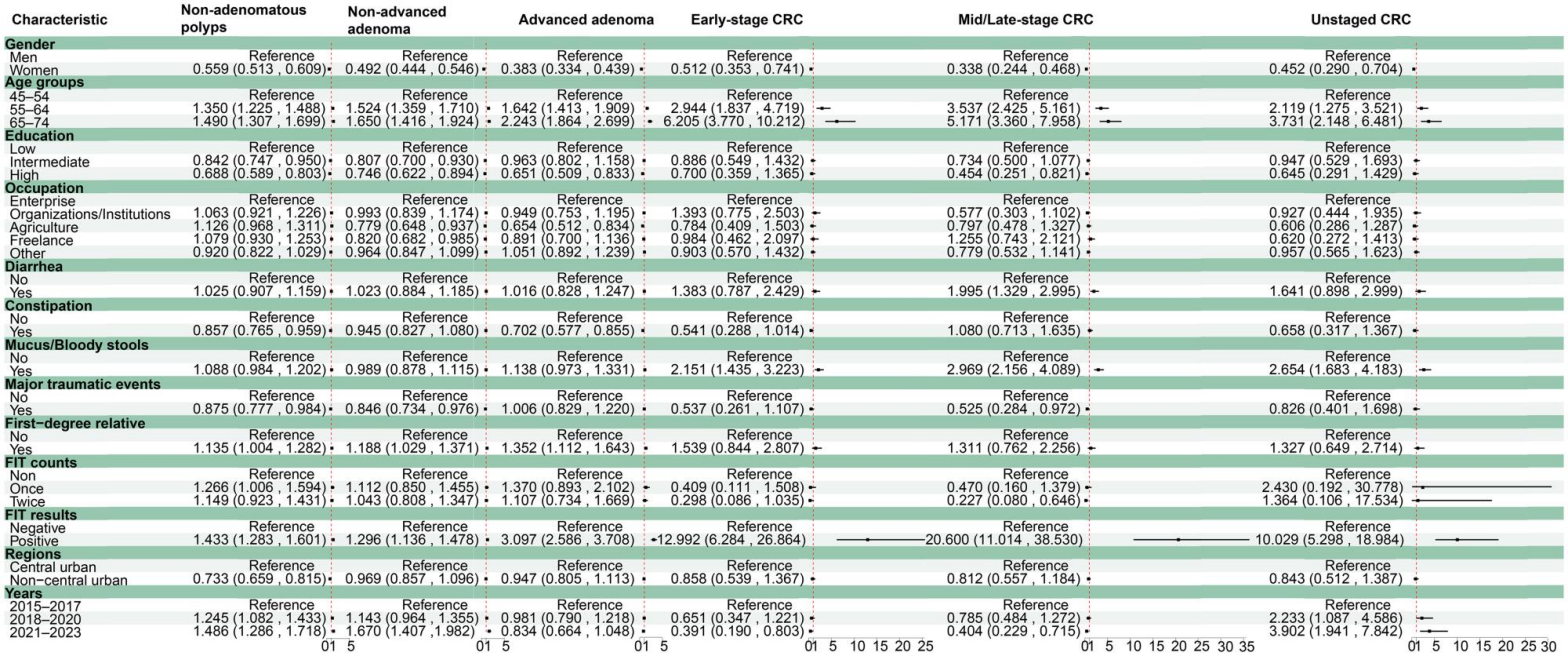
Multinomial logistic regression of factors influencing the detection of colorectal lesions by colonoscopy. Analyses were weighted by sex and age.

We conducted multinomial logistic model analysis using a forward stepwise selection approach. This was done to explore the potential factors associated with different types of colorectal lesions detected by colonoscopy (Fig. 3). Women were negatively associated with all categories of colorectal lesions. Older age and a positive FIT result were consistently positively associated with all lesion types. Diarrhea was positively associated only with mid/late-stage CRC (OR = 1.995, 95% CI: 1.329–2.995). Constipation was negatively related to non-adenomatous polyps (OR = 0.857, 95% CI: 0.765–0.959) and advanced adenomas (OR = 0.702, 95% CI: 0.577–0.855). But it showed no significant associations with other lesion types. Mucus/bloody stools were not associated with polypoid or adenomatous lesions. However, it showed a positive association with CRC, including early-stage CRC (OR = 2.151, 95% CI: 1.435–3.223), mid/late-stage CRC (OR = 2.969, 95% CI: 2.156–4.089), and unstaged CRC (OR = 2.654, 95% CI: 1.683–4.183). A family history of CRC in FDRs was also positively associated with non-adenomatous polyps (OR = 1.135, 95% CI: 1.004–1.282), non-advanced adenomas (OR = 1.188, 95% CI: 1.029–1.371), and advanced adenomas (OR = 1.352, 95% CI: 1.112–1.643), but not with other lesion categories. Residential region showed a negative association with non-adenomatous polyps (OR = 0.733, 95% CI: 0.659–0.815).

In addition, both the *χ*^2^ test (Supplementary Table 4) and multinomial logistic regression analyses revealed an increasing trend in the detection rates of low-risk lesions (non-adenomatous polyps and non-advanced adenomas). Additionally, it showed a significant decline in the detection of high-risk lesions (early-stage and mid/late-stage CRC) compared with the first screening round (2015–2017). Therefore, these findings suggest that repeated screenings were more effective in identifying early lesions and potentially preventing progression to CRC.

### Follow-up of the initial screening population

As of 30 June 2024, 3,456 CRC cases were identified among all screened participants through active and passive follow-up. Of these, 1,630 cases (47.16%) occurred among individuals with positive initial screening results. In contrast, 1,826 cases (52.84%) were found among those with negative results.

In the screening-positive group, 22,157 individuals underwent colonoscopy within the program-defined 10-month follow-up period. Among them, 915 CRC cases were detected via colonoscopy and classified as TP. In contrast, 9,134 participants who underwent colonoscopy were found to have no CRC and were categorized as FP. An additional 715 CRC cases were identified through passive follow-up among the 61,433 screening-positive individuals who did not undergo colonoscopy. Of these, 495 cases were diagnosed within 12 months of the initial screening result—comprising 151 early-stage, 247 mid/late-stage, and 97 unstaged CRC. However, these 495 cases were not included in test performance calculations due to the lack of a confirmatory colonoscopy within the defined timeframe (Table S5).

Among the 492,253 individuals in the screening-negative group, 1,826 CRC cases were identified via cancer registry linkage. These included 366 early-stage, 892 mid/late-stage, and 568 unstageable cases. Of these, 233 CRC cases were diagnosed within 1 year of the initial screening and were classified as FN for test performance evaluation. The remaining 492,020 participants had no CRC diagnosis within 1 year and were considered TN. Furthermore, CRC cases diagnosed beyond a 1-year timeframe were considered part of long-term follow-up and were not included in sensitivity and specificity calculations (Table S5).

## Discussion

This study provides a comprehensive evaluation of a large-scale, population-based CRC screening program implemented in Guangzhou, China, between 2015 and 2023. Among the 575,843 participants who underwent initial screening, 83,590 (14.52%; weighted, 14.55%) were identified as high-risk for CRC based on positive screening results. However, only 26.51% (weighted, 28.17%) of these high-risk individuals completed the recommended follow-up colonoscopy. This relatively low adherence rate highlights a critical gap in the CRC screening pathway—namely, that the benefits of early detection and prevention may be substantially undermined when high-risk individuals fail to undergo colonoscopy.

Factors such as age, education, previous anorectal symptoms, history of colon cancer in FDRs, number and results of FIT were all associated with the adherence rate of colonoscopy. Among those who underwent colonoscopy, the weighted detection rates of non-advanced adenoma, advanced adenoma, and CRC were 16.58%, 8.74%, and 3.04%, respectively. Notably, these detection rates were higher in men than in women and showed an increasing trend with age. Multivariate analysis also indicated that men, older age, and positive FIT results were associated with the presence of all types of colorectal lesions.

Screening effectively reduces CRC incidence and mortality, primarily attributed to colonoscopy. As the gold standard for CRC screening, colonoscopy enables early detection and facilitates the removal of precancerous polyps. However, low adherence to colonoscopy remains a persistent challenge. Most participants with positive initial screening results do not complete a colonoscopy, leaving potential adenomas or early-stage CRC undiagnosed. Consequently, this undermines the overall effectiveness of CRC screening. Compared with other domestic and international programs, the colonoscopy adherence observed in our study was suboptimal. Randomized trials conducted in four countries (Poland, Norway, Sweden, and the Netherlands) have reported varying adherence rates to colonoscopy, ranging from 33.00% in Poland to 60.70% in Norway, with an average adherence of 42.00% [21]. In comparison, the adherence rate observed in the Guangzhou CRC screening program was lower than those in Australia (70.00%), Korea (46.60%), Beijing (55.44%), Shanghai (39.83%), and Tianjin (27.10%) [14, 22–25], but higher than those in Chongqing (19.02%) and the national population-based colonoscopy screening program in China (14.00%) [13, 26]. Notably, Chinese cities such as Beijing, Tianjin, and Shanghai offer free colonoscopy screening, which partly explains the higher adherence observed in those regions. Consistent with this, our findings indicated that adherence was significantly higher in the years and districts of Guangzhou where financial allowances were provided than in those without such allowances. Notably, this underscores the potential benefit of reducing out-of-pocket costs to improve participation in colonoscopy screening.

The results of this study indicated that colonoscopy adherence decreased with increasing age, consistent with previous research [14, 26]. This trend may be attributed to a higher prevalence of comorbidities, such as cardiovascular disease and diabetes, among older adults, which increases concerns regarding colonoscopy preparation and the potential risk associated with colonoscopy [27]. Additionally, the discomfort of the bowel cleansing process and anxiety about the procedure, especially among older adults who have not undergone such tests before, further reduce adherence [28]. To alleviate the discomfort associated with bowel preparation, strategies aimed at simplifying bowel preparation may be effective. For example, Kang et al. [29] demonstrated that same-day, single-dose, low-volume (SSL) administration (taking 2-L purgative 4–6 hours before colonoscopy) was as effective as the standard split-dose method (taking 2-L laxative in the evening before, between 19:00 and 21:00). Importantly, this significantly reducing adverse effects such as nausea, vomiting, and abdominal pain [29]. Moreover, a greater proportion of patients in the SSL group expressed willingness to undergo repeat colonoscopy. These findings suggest that optimizing bowel preparation protocols may improve adherence and facilitate broader implementation of colonoscopy-based screening programs.

In addition, our findings revealed that a higher educational level was significantly associated with increased colonoscopy adherence. Individuals with higher education levels may possess greater health literacy and awareness of the benefits of early detection, enabling them to understand screening guidelines better and interpret medical information. They are also more likely to seek preventive healthcare services and respond proactively to screening invitations [30, 31]. Additionally, higher adherence rates were observed among individuals with chronic diarrhea, chronic constipation, and mucus/bloody stools. These symptoms are both physically and psychologically distressing, potentially increasing health vigilance. According to the Health Belief Model, individuals who perceive themselves as being at higher risk for disease are more likely to engage in preventive behaviors, including cancer screening and routine health examinations [32, 33]. Similarly, individuals with a family history of CRC also demonstrated higher adherence to colonoscopy, consistent with the results of previous literature [34]. This may be attributed to heightened perceived susceptibility, greater risk awareness, and stronger recognition of the importance of early detection among FDRs of CRC patients [35]. These individuals may also be more receptive to physicians’ recommendations—a factor shown to strongly influence CRC screening uptake in Chinese populations [36]. Furthermore, exposure to cancer-related experiences within the family may also enhance their motivation and create a more supportive environment for screening participation. In contrast, individuals with a history of non-colorectal cancers were less likely to complete a colonoscopy. This reluctance may stem from their fear of procedural anxiety or fear of discovering new malignancies [37, 38]. For this group, peer education, psychological counseling, and clear communication about the safety and importance of colonoscopy could be instrumental in improving participation rates [39, 40].

This study reaffirmed several well-established predictors of colorectal lesions. Men [41] and older age [42] were associated with higher detection rates of adenomas, advanced adenomas, and early-stage cancers, underscoring their importance in risk stratification. A positive FIT result was strongly associated with all lesion types, supporting the robustness of the two-step screening strategy that combines symptom-based and fecal test methods. Notably, gastrointestinal symptoms demonstrated varying associations with lesion severity. Chronic diarrhea was also significantly associated with mid/late-stage CRC, suggesting it may serve as a late-stage warning sign. Mucus/bloody stools were not linked to polyps or adenomas but were strongly predictive of CRC at all stages, highlighting their potential as early indicators of malignancy. Conversely, constipation showed a negative association with specific benign lesions and no association with CRC, indicating limited utility in risk prediction. Furthermore, lesion detection patterns evolved. Compared with the 2015–2017 cohort, those screened in 2021–2023 had a higher detection rate of low-risk lesions and a lower rate of advanced CRC. Thus, this shift implies improved early detection and points to the cumulative benefits of sustained program implementation.

The implementation of the CRC screening program in Guangzhou yielded several valuable insights for future public health efforts. A key strength of this study lies in the establishment of one of the largest and longest-running community-based CRC screening datasets in South China. Data were collected in a standardized manner by trained professionals and centrally managed through the Guangzhou CRC Screening Registry System. This ensured data integrity and supported long-term evaluation. In particular, the integration of digital tools—most notably the “Changtan” applet—represents an innovative feature of this program. It enabled real-time data entry and retrieval, thereby enhancing information transparency and facilitating active engagement from participants and healthcare providers. Furthermore, the study demonstrated the importance of community mobilization strategies. Collaborations with neighborhood committees and the use of diverse outreach channels—including posters, videos, and educational materials—contributed to improved public awareness and increased participation. This was particularly true among traditionally under-screened populations. Moreover, the application of population weighting based on the 2020 Guangzhou census enhanced the representativeness of the findings. This methodological refinement allowed for more accurate estimation of screening performance indicators and subgroup disparities. Taken together, the integration of digital tools, structured data infrastructure, and community-based mobilization presents a compelling and scalable model for the implementation of large-scale CRC screening programs within similar urban public health systems in China.

### Limitations and disadvantages

Our study has several limitations that should be acknowledged. First, the study population was not derived from random sampling but recruited through structured, community-based mobilization under district-level quotas set by local health authorities. This non-probabilistic recruitment may introduce selection bias, as participants could differ from the general population in health awareness, socioeconomic status, or access to healthcare. A notable example is the gender imbalance observed in our cohort, with women comprising a disproportionately large share of participants. This pattern, commonly reported in community-based screening programs, likely reflects gender differences in health-seeking behavior rather than a flaw in recruitment strategy. To mitigate potential bias, we applied post-stratification weighting based on age and gender-specific distributions from the 2020 Guangzhou Census. Importantly, this recruitment strategy is consistent with those used in other national CRC screening programs across China, supporting the comparability and relevance of our findings within the broader public health context.

Second, while we estimated an overall participation rate of 10.87% among individuals aged 45–74 years using census and screening records, individual-level data on invitations and responses were not systematically recorded across all districts. Consequently, this limited our ability to analyze participation patterns and identify determinants of engagement. Moreover, the lack of information on reasons for non-participation hindered further exploration of barriers to uptake. To address this, future program phases will incorporate electronic tracking systems to improve the monitoring of invitation-response workflows and facilitate the development of targeted outreach strategies.

Third, adherence to follow-up colonoscopy among screening-positive individuals remained suboptimal. This compromised the effectiveness of the program by potentially delaying the diagnosis of colorectal lesions. Consistent with previous studies [14], we also found that some residents preferred to undergo colonoscopy at higher-level non-designated hospitals, resulting in incomplete colonoscopy data collection and affecting overall outcome assessment. This preference may stem from perceptions of greater trust or convenience associated with these institutions. To address this, expanding the network of sentinel hospitals to include more accessible and trusted high-level facilities may improve both service coverage and patient trust.

Finally, this study employed a qualitative FIT for initial screening. Although cost-effective and widely available, it lacks the adjustable positivity thresholds and higher reproducibility offered by quantitative FIT. Considerations of feasibility, affordability, and alignment with national screening guidelines and expert consensus at the time of program initiation drove the selection of qualitative FIT. However, we acknowledge that the use of quantitative FIT could enable more precise risk stratification and better programmatic control. As part of our ongoing efforts to optimize the screening strategy, we are assessing the potential integration of quantitative FIT in future rounds through pilot studies. In doing so, we are evaluating its performance, cost-effectiveness, and operational feasibility in community-based settings.

In summary, this study presents a real-world evaluation of a large-scale, community-based CRC screening program conducted in Guangzhou, China, from 2015 to 2023. Based on data from 575,843 participants, the findings demonstrate the feasibility of implementing a two-step screening strategy—risk assessment combined with FIT—in routine practice. Notably, detection rates for adenomas and CRC were higher among older individuals, men, and those with positive FIT results or specific symptoms. However, suboptimal adherence to follow-up colonoscopy among screening-positive individuals remains a significant barrier to program effectiveness. Screening participation was influenced by personal factors such as age, education level, and symptom presence, as well as systemic issues like financial burden and healthcare access. As such, this underscores the need to address both individual-level and healthcare access limitations to improve uptake. To enhance screening impact, structural improvements, including electronic tracking of invitations, broader coverage of high-level hospitals, and optimized bowel preparation protocols, are warranted. Additionally, the integration of digital tools and community-based outreach strategies showed promise in improving participation. Despite limitations such as non-randomized recruitment and incomplete follow-up data, this study provides substantial evidence on program performance and behavioral determinants. Thus, it offers insights to inform CRC screening strategies in China and other resource-limited settings.

## Supplementary Material

goag011_Supplementary_Data

## Data Availability

Data are available upon reasonable request. The datasets used or analyzed during the current study are available from the corresponding author upon reasonable request.
