# Supplementary material for: Current status and challenges of colorectal cancer screening program in Guangzhou, China: a population-based study
Source: Gastroenterol Rep (Oxf). 2026 May 19;14:goag011. doi: 10.1093/gastro/goag011 (PMC13183681; doi:10.1093/gastro/goag011)
Supplement: goag011_Supplementary_Data [file goag011_supplementary_data.zip › 2025-237 Supplementary Tables 1-5.docx]

**Supplementary Table 1.** Sex-age weighted according to 2020 Guangzhou Municipal Census data

| **Age group**  **(years)** | **Population composition of Guangzhou in 2020** | | | | **Population composition of this study** | | | | **Weighting factor** | |
| --- | --- | --- | --- | --- | --- | --- | --- | --- | --- | --- |
|  | **Men** | | **Women** | | **Men** | | **Women** | | **Men** | **Women** |
|  | ***N*** | **Proportion** | ***N*** | **Proportion** | ***N*** | **Proportion** | ***N*** | **Proportion** |  |  |
| 45–54 | 1,425,443 | 0.144,5 | 1,289,489 | 0.146,4 | 40,686 | 0.070,7 | 80,399 | 0.139,6 | 2.04 | 1.05 |
| 55–64 | 837,860 | 0.084,9 | 810,657 | 0.092,0 | 77,043 | 0.133,8 | 138,689 | 0.240,8 | 0.63 | 0.38 |
| 65–74 | 445,159 | 0.045,1 | 489,749 | 0.055,6 | 101,217 | 0.175,8 | 137,809 | 0.239,3 | 0.26 | 0.23 |

**Supplementary Table 2.** Multicollinearity assessment of factors associated with colonoscopy adherence

| **Characteristic** | **Unstandardized coefficients** | | **Standardized coefficients** | ***t*** | ***P* values** | **Collinearity statistics** | |
| --- | --- | --- | --- | --- | --- | --- | --- |
|  | **B** | **Std. Error** | **Beta** |  |  | **Tolerance** | **VIF** |
| (Constant) | 0.035 | 0.022 |  | 1.579 | 0.114 |  |  |
| Gender | 0.006 | 0.004 | 0.007 | 1.478 | 0.140 | 0.937 | 1.068 |
| Age group | −0.028 | 0.003 | −0.048 | −10.003 | <0.001 | 0.888 | 1.126 |
| Region | 0.008 | 0.005 | 0.008 | 1.654 | 0.098 | 0.895 | 1.117 |
| Marital status | −0.016 | 0.008 | −0.009 | −1.949 | 0.051 | 0.953 | 1.049 |
| Education | 0.034 | 0.003 | 0.049 | 9.908 | <0.001 | 0.813 | 1.230 |
| Occupation | −0.001 | 0.001 | −0.005 | −1.066 | 0.286 | 0.935 | 1.070 |
| Allowance | 0.031 | 0.006 | 0.024 | 5.113 | <0.001 | 0.925 | 1.081 |
| Diarrhea | 0.116 | 0.007 | 0.084 | 17.210 | <0.001 | 0.850 | 1.177 |
| Constipation | 0.058 | 0.006 | 0.050 | 9.887 | <0.001 | 0.795 | 1.257 |
| Mucus/Bloody stools | 0.073 | 0.006 | 0.074 | 12.699 | <0.001 | 0.587 | 1.703 |
| Appendicitis | −0.009 | 0.008 | −0.005 | −1.158 | 0.247 | 0.894 | 1.118 |
| Cholecystitis | 0.001 | 0.008 | 0.001 | 0.121 | 0.903 | 0.897 | 1.115 |
| Non-colon cancer | −0.018 | 0.009 | −0.010 | −1.938 | 0.053 | 0.688 | 1.453 |
| Major traumatic events | 0.027 | 0.006 | 0.023 | 4.618 | <0.001 | 0.834 | 1.199 |
| First-degree relative | 0.075 | 0.007 | 0.068 | 10.293 | <0.001 | 0.461 | 2.167 |
| FOBT count | 0.019 | 0.004 | 0.026 | 4.592 | <0.001 | 0.612 | 1.633 |
| FOBT results | 0.207 | 0.008 | 0.228 | 24.469 | <0.001 | 0.233 | 4.301 |
| Questionnaire | 0.016 | 0.011 | 0.017 | 1.490 | 0.136 | 0.154 | 6.500 |
| Years | 0.005 | 0.003 | 0.009 | 1.524 | 0.127 | 0.580 | 1.724 |

Std. = standard, VIF = variance inflation factor.

**Supplementary Table 3.** Multicollinearity diagnosis of factors associated with lesion detection in colonoscopy

| **Characteristic** | | | **Unstandardized coefficients** | | | | **Standardized coefficients** | | ***t*** | | ***P* values** | | **Collinearity statistics** | | |
| --- | --- | --- | --- | --- | --- | --- | --- | --- | --- | --- | --- | --- | --- | --- | --- |
|  |  |  | **B** | | **Std. Error** | | **Beta** | |  |  |  |  | **Tolerance** | | **VIF** |
| (Constant) | 1.743 | | 0.288 | |  | | 6.052 | | <0.001 | |  | |  | |  |
| Gender | 0.351 | | 0.020 | | 0.146 | | 17.203 | | <0.001 | | 0.957 | | 1.045 | |  |
| Age group | −0.134 | | 0.011 | | −0.100 | | −11.769 | | <0.001 | | 0.956 | | 1.046 | |  |
| Marital status | −0.019 | | 0.042 | | −0.004 | | −0.459 | | 0.646 | | 0.942 | | 1.061 | |  |
| Education | 0.020 | | 0.013 | | 0.013 | | 1.499 | | 0.134 | | 0.936 | | 1.068 | |  |
| Occupation | 0.025 | | 0.007 | | 0.029 | | 3.342 | | 0.001 | | 0.925 | | 1.081 | |  |
| Diarrhea | −0.056 | | 0.029 | | −0.017 | | −1.901 | | 0.057 | | 0.896 | | 1.116 | |  |
| Constipation | 0.068 | | 0.027 | | 0.022 | | 2.545 | | 0.011 | | 0.888 | | 1.126 | |  |
| Mucus/Bloody stools | −0.132 | | 0.024 | | −0.052 | | −5.502 | | <0.001 | | 0.785 | | 1.273 | |  |
| Appendicitis | 0.008 | | 0.040 | | 0.002 | | 0.197 | | 0.844 | | 0.966 | | 1.035 | |  |
| Cholecystitis | 0.005 | | 0.042 | | 0.001 | | 0.129 | | 0.897 | | 0.964 | | 1.037 | |  |
| Non-colon cancer | −0.111 | | 0.051 | | −0.019 | | −2.153 | | 0.031 | | 0.907 | | 1.102 | |  |
| Major traumatic events | 0.057 | | 0.029 | | 0.018 | | 1.989 | | 0.047 | | 0.878 | | 1.139 | |  |
| First-degree relative | −0.115 | | 0.030 | | −0.038 | | −3.863 | | <0.001 | | 0.707 | | 1.415 | |  |
| FOBT count | −0.088 | | 0.022 | | −0.042 | | −4.044 | | <0.001 | | 0.650 | | 1.539 | |  |
| FOBT results | −0.468 | | 0.027 | | −0.195 | | −17.333 | | <0.001 | | 0.546 | | 1.832 | |  |
| Region | 0.040 | | 0.023 | | 0.015 | | 1.720 | | 0.085 | | 0.885 | | 1.130 | |  |
| Years | −0.008 | | 0.016 | | −0.005 | | −0.529 | | 0.597 | | 0.665 | | 1.504 | |  |

Std. = standard, VIF = variance inflation factor.

**Supplementary Table 4**. *χ^2^* test for the detection rates of various lesions under colonoscopy

| **Characteristic** | **Non-adenomatous**  **polyps** | | **Non-advanced**  **adenomas** | | **Advanced**  **adenomas** | | **Early-stage**  **CRC** | | **Mid/Late-stage**  **CRC** | | **Unstaged**  **CRC** | |
| --- | --- | --- | --- | --- | --- | --- | --- | --- | --- | --- | --- | --- |
|  | **Cases (*N*)** | **DR (%)** | **Cases (*N*)** | **DR (%)** | **Cases (*N*)** | **DR (%)** | **Cases (*N*)** | **DR (%)** | **Cases (*N*)** | **DR (%)** | **Cases (*N*)** | **DR (%)** |
| **Gender** |  |  |  |  |  |  |  |  |  |  |  |  |
| Men | 2,113 | 30.55 | 1,317 | 19.04 | 760 | 10.98 | 76 | 1.10 | 130 | 1.88 | 59 | 0.85 |
| Women | 1,634 | 25.12 | 909 | 13.97 | 413 | 6.35 | 52 | 0.80 | 59 | 0.90 | 33 | 0.51 |
| ***χ^2^*** | 49.247 |  | 62.316 |  | 90.535 |  | 3.189 |  | 22.863 |  | 5.891 |  |
| ***P*** | <0.001 |  | <0.001 |  | <0.001 |  | 0.074 |  | <0.001 |  | 0.015 |  |
| **Age groups** |  |  |  |  |  |  |  |  |  |  |  |  |
| 45–54 | 1,945 | 27.10 | 1,107 | 15.42 | 488 | 6.80 | 30 | 0.42 | 46 | 0.65 | 36 | 0.50 |
| 55–64 | 1,215 | 28.70 | 761 | 17.97 | 429 | 10.14 | 51 | 1.19 | 87 | 2.06 | 30 | 0.72 |
| 65–74 | 587 | 29.20 | 358 | 17.80 | 255 | 12.70 | 47 | 2.34 | 55 | 2.75 | 25 | 1.26 |
| ***χ^2^*** | 5.274 |  | 15.044 |  | 83.413 |  | 65.373 |  | 69.020 |  | 12.909 |  |
| ***P*** | 0.072 |  | 0.001 |  | <0.001 |  | <0.001 |  | <0.001 |  | 0.002 |  |
| **Regions** |  |  |  |  |  |  |  |  |  |  |  |  |
| Central urban area | 2,682 | 28.42 | 1,526 | 16.17 | 836 | 8.86 | 95 | 1.00 | 133 | 1.41 | 62 | 0.66 |
| Non-central urban area | 1,066 | 26.74 | 700 | 17.56 | 337 | 8.45 | 33 | 0.83 | 56 | 1.40 | 30 | 0.75 |
| ***χ^2^*** | 3.881 |  | 3.944 |  | 0.568 |  | 0.946 |  | 0.000 |  | 0.378 |  |
| ***P*** | 0.049 |  | 0.047 |  | 0.451 |  | 0.331 |  | 0.985 |  | 0.539 |  |
| **Marital status** |  |  |  |  |  |  |  |  |  |  |  |  |
| Married | 3,522 | 28.02 | 2,099 | 16.70 | 1,105 | 8.79 | 121 | 0.97 | 181 | 1.44 | 85 | 0.68 |
| Single/Divorced/Widowed | 225 | 26.47 | 126 | 14.85 | 67 | 7.93 | 6 | 0.72 | 8 | 0.89 | 6 | 0.76 |
| ***χ^2^*** | 0.946 |  | 2.021 |  | 0.823 |  | 0.560 |  | 1.434 |  | 0.010 |  |
| ***P*** | 0.331 |  | 0.155 |  | 0.364 |  | 0.454 |  | 0.231 |  | 0.919 |  |
| **Education** |  |  |  |  |  |  |  |  |  |  |  |  |
| Low | 749 | 29.52 | 438 | 17.24 | 222 | 8.74 | 30 | 1.20 | 52 | 2.03 | 19 | 0.73 |
| Intermediate | 2,218 | 28.04 | 1,278 | 16.15 | 755 | 9.55 | 76 | 0.96 | 116 | 1.47 | 57 | 0.73 |
| High | 780 | 26.22 | 510 | 17.15 | 195 | 6.57 | 21 | 0.72 | 21 | 0.71 | 16 | 0.53 |

**Supplementary Table 4 (Continued)**

| **Characteristic** | | **Non-adenomatous**  **polyps** | | | | **Non-advanced**  **adenomas** | | | | **Advanced**  **adenomas** | | | | **Early-stage**  **CRC** | | | | **Mid/Late-stage**  **CRC** | | | | **Unstaged**  **CRC** | | | |
| --- | --- | --- | --- | --- | --- | --- | --- | --- | --- | --- | --- | --- | --- | --- | --- | --- | --- | --- | --- | --- | --- | --- | --- | --- | --- |
|  |  | **Cases (*N*)** | | **DR* (%)** | | **Cases (*N*)** | | **DR* (%)** | | **Cases (*N*)** | | **DR* (%)** | | **Cases (*N*)** | | **DR* (%)** | | **Cases (*N*)** | | **DR* (%)** | | **Cases (*N*)** | | **DR* (%)** | |
| ***χ^2^*** | 7.489 | |  | | 2.535 | |  | | 24.250 | |  | | 3.353 | |  | | 18.241 | |  | | 1.245 | |  | |  |
| ***P*** | 0.024 | |  | | 0.282 | |  | | <0.001 | |  | | 0.187 | |  | | <0.001 | |  | | 0.537 | |  | |  |
| **Occupation** |  | |  | |  | |  | |  | |  | |  | |  | |  | |  | |  | |  | |  |
| Enterprise | 1,060 | | 28.04 | | 662 | | 17.53 | | 347 | | 9.18 | | 37 | | 0.97 | | 59 | | 1.56 | | 31 | | 0.83 | |  |
| Organizations/Institutions | 506 | | 27.42 | | 308 | | 16.71 | | 132 | | 7.17 | | 20 | | 1.07 | | 12 | | 0.67 | | 11 | | 0.57 | |  |
| Agriculture | 625 | | 31.43 | | 307 | | 15.45 | | 145 | | 7.27 | | 19 | | 0.96 | | 35 | | 1.76 | | 13 | | 0.63 | |  |
| Freelance | 457 | | 30.91 | | 227 | | 15.32 | | 114 | | 7.69 | | 9 | | 0.62 | | 22 | | 1.51 | | 8 | | 0.51 | |  |
| Other | 1,100 | | 25.39 | | 721 | | 16.66 | | 435 | | 10.05 | | 43 | | 0.99 | | 60 | | 1.39 | | 30 | | 0.69 | |  |
| ***χ^2^*** | 32.676 | |  | | 5.950 | |  | | 23.213 | |  | | 2.296 | |  | | 10.166 | |  | | 1.685 | |  | |  |
| ***P*** | <0.001 | |  | | 0.203 | |  | | <0.001 | |  | | 0.681 | |  | | 0.038 | |  | | 0.793 | |  | |  |
| **Diarrhea** |  | |  | |  | |  | |  | |  | |  | |  | |  | |  | |  | |  | |  |
| No | 3,200 | | 28.09 | | 1,902 | | 16.69 | | 1,034 | | 9.08 | | 112 | | 0.98 | | 155 | | 1.36 | | 78 | | 0.68 | |  |
| Yes | 547 | | 26.94 | | 324 | | 15.95 | | 138 | | 6.82 | | 16 | | 0.78 | | 34 | | 1.68 | | 14 | | 0.69 | |  |
| ***χ^2^*** | 1.152 | |  | | 0.691 | |  | | 11.231 | |  | | 0.697 | |  | | 1.225 | |  | | 0.001 | |  | |  |
| ***P*** | 0.283 | |  | | 0.406 | |  | | 0.001 | |  | | 0.404 | |  | | 0.268 | |  | | 0.982 | |  | |  |
| **Constipation** |  | |  | |  | |  | |  | |  | |  | |  | |  | |  | |  | |  | |  |
| No | 3,113 | | 28.66 | | 1,822 | | 16.77 | | 1,029 | | 9.48 | | 116 | | 1.07 | | 159 | | 1.46 | | 83 | | 0.77 | |  |
| Yes | 635 | | 24.77 | | 404 | | 15.77 | | 144 | | 5.61 | | 11 | | 0.44 | | 30 | | 1.19 | | 8 | | 0.33 | |  |
| ***χ^2^*** | 15.501 | |  | | 1.504 | |  | | 38.616 | |  | | 9.017 | |  | | 1.278 | |  | | 6.283 | |  | |  |
| ***P*** | <0.001 | |  | | 0.220 | |  | | <0.001 | |  | | 0.003 | |  | | 0.258 | |  | | 0.012 | |  | |  |
| **Mucus/Bloody stools** |  | |  | |  | |  | |  | |  | |  | |  | |  | |  | |  | |  | |  |
| No | 2,514 | | 27.89 | | 1,512 | | 16.77 | | 854 | | 9.47 | | 87 | | 0.97 | | 109 | | 1.21 | | 53 | | 0.59 | |  |
| Yes | 1,233 | | 27.98 | | 714 | | 16.20 | | 319 | | 7.24 | | 40 | | 0.92 | | 80 | | 1.82 | | 38 | | 0.87 | |  |
| ***χ^2^*** | 0.015 | |  | | 0.683 | |  | | 18.454 | |  | | 0.102 | |  | | 7.857 | |  | | 3.319 | |  | |  |
| ***P*** | 0.903 | |  | | 0.409 | |  | | <0.001 | |  | | 0.749 | |  | | 0.005 | |  | | 0.069 | |  | |  |
| **Appendicitis** |  | |  | |  | |  | |  | |  | |  | |  | |  | |  | |  | |  | |  |

**Supplementary Table 4 (Continued)**

| **Characteristic** | | **Non-adenomatous**  **polyps** | | | | **Non-advanced**  **adenomas** | | | | **Advanced**  **adenomas** | | | | **Early-stage**  **CRC** | | | | **Mid/Late-stage**  **CRC** | | | | **Unstaged**  **CRC** | | | |
| --- | --- | --- | --- | --- | --- | --- | --- | --- | --- | --- | --- | --- | --- | --- | --- | --- | --- | --- | --- | --- | --- | --- | --- | --- | --- |
|  |  | **Cases (*N*)** | | **DR* (%)** | | **Cases (*N*)** | | **DR* (%)** | | **Cases (*N*)** | | **DR* (%)** | | **Cases (*N*)** | | **DR* (%)** | | **Cases (*N*)** | | **DR* (%)** | | **Cases (*N*)** | | **DR* (%)** | |
| No | 3,488 | | 27.95 | | 2,063 | | 16.53 | | 1,113 | | 8.92 | | 122 | | 0.98 | | 178 | | 1.43 | | 87 | | 0.70 | |  |
| Yes | 259 | | 27.49 | | 163 | | 17.24 | | 60 | | 6.34 | | 6 | | 0.60 | | 11 | | 1.12 | | 5 | | 0.50 | |  |
| ***χ^2^*** | 0.104 | |  | | 0.342 | |  | | 7.233 | |  | | 1.088 | |  | | 0.431 | |  | | 0.362 | |  | |  |
| ***P*** | 0.748 | |  | | 0.559 | |  | | 0.007 | |  | | 0.297 | |  | | 0.511 | |  | | 0.547 | |  | |  |
| **Cholecystitis** |  | |  | |  | |  | |  | |  | |  | |  | |  | |  | |  | |  | |  |
| No | 3,521 | | 27.94 | | 2,094 | | 16.61 | | 1,108 | | 8.79 | | 121 | | 0.96 | | 184 | | 1.46 | | 90 | | 0.71 | |  |
| Yes | 226 | | 27.62 | | 132 | | 16.13 | | 65 | | 7.97 | | 6 | | 0.76 | | 5 | | 0.59 | | 2 | | 0.19 | |  |
| ***χ^2^*** | 0.037 | |  | | 0.126 | |  | | 0.687 | |  | | 0.421 | |  | | 3.984 | |  | | 2.488 | |  | |  |
| ***P*** | 0.848 | |  | | 0.722 | |  | | 0.407 | |  | | 0.517 | |  | | 0.046 | |  | | 0.115 | |  | |  |
| **Non-colon cancer** |  | |  | |  | |  | |  | |  | |  | |  | |  | |  | |  | |  | |  |
| No | 3,580 | | 27.88 | | 2,138 | | 16.65 | | 1,133 | | 8.82 | | 126 | | 0.98 | | 186 | | 1.45 | | 87 | | 0.68 | |  |
| Yes | 167 | | 28.75 | | 87 | | 15.01 | | 40 | | 6.86 | | 2 | | 0.32 | | 2 | | 0.43 | | 4 | | 0.74 | |  |
| ***χ^2^*** | 0.205 | |  | | 1.130 | |  | | 2.621 | |  | | 2.388 | |  | | 4.908 | |  | | 9.470 | |  | |  |
| ***P*** | 0.651 | |  | | 0.288 | |  | | 0.105 | |  | | 0.122 | |  | | 0.027 | |  | | 0.002 | |  | |  |
| **Major traumatic events** |  | |  | |  | |  | |  | |  | |  | |  | |  | |  | |  | |  | |  |
| No | 3,193 | | 28.42 | | 1,902 | | 16.93 | | 1,018 | | 9.06 | | 119 | | 1.06 | | 177 | | 1.58 | | 83 | | 0.74 | |  |
| Yes | 554 | | 25.34 | | 323 | | 14.78 | | 155 | | 7.08 | | 8 | | 0.38 | | 12 | | 0.53 | | 9 | | 0.40 | |  |
| ***χ^2^*** | 8.678 | |  | | 6.136 | |  | | 8.941 | |  | | 9.384 | |  | | 13.903 | |  | | 2.880 | |  | |  |
| ***P*** | 0.003 | |  | | 0.013 | |  | | 0.003 | |  | | 0.002 | |  | | <0.001 | |  | | 0.090 | |  | |  |
| **First-degree relative** |  | |  | |  | |  | |  | |  | |  | |  | |  | |  | |  | |  | |  |
| No | 3,011 | | 27.94 | | 1,755 | | 16.28 | | 977 | | 9.06 | | 114 | | 1.05 | | 173 | | 1.60 | | 82 | | 0.76 | |  |
| Yes | 736 | | 27.85 | | 470 | | 17.80 | | 196 | | 7.41 | | 14 | | 0.53 | | 16 | | 0.61 | | 10 | | 0.36 | |  |
| ***χ^2^*** | 0.008 | |  | | 3.452 | |  | | 7.263 | |  | | 6.271 | |  | | 15.280 | |  | | 4.565 | |  | |  |
| ***P*** | 0.927 | |  | | 0.063 | |  | | 0.007 | |  | | 0.012 | |  | | <0.001 | |  | | 0.033 | |  | |  |
| **FOBT counts** |  | |  | |  | |  | |  | |  | |  | |  | |  | |  | |  | |  | |  |
| Non | 138 | | 22.94 | | 92 | | 15.28 | | 29 | | 4.82 | | 4 | | 0.59 | | 5 | | 0.91 | | 1 | | 0.11 | |  |

**Supplementary Table 4 (Continued)**

| **Characteristic** | | **Non-adenomatous**  **polyps** | | | | **Non-advanced**  **adenomas** | | | | **Advanced**  **adenomas** | | | | **Early-stage**  **CRC** | | | | **Mid/Late-stage**  **CRC** | | | | **Unstaged**  **CRC** | | | |
| --- | --- | --- | --- | --- | --- | --- | --- | --- | --- | --- | --- | --- | --- | --- | --- | --- | --- | --- | --- | --- | --- | --- | --- | --- | --- |
|  |  | **Cases (*N*)** | | **DR* (%)** | | **Cases (*N*)** | | **DR* (%)** | | **Cases (*N*)** | | **DR* (%)** | | **Cases (*N*)** | | **DR* (%)** | | **Cases (*N*)** | | **DR* (%)** | | **Cases (*N*)** | | **DR* (%)** | |
| Once | 1,667 | | 30.34 | | 1,000 | | 18.19 | | 420 | | 7.65 | | 32 | | 0.59 | | 68 | | 1.24 | | 59 | | 1.08 | |  |
| Twice | 1,942 | | 26.51 | | 1,134 | | 15.48 | | 723 | | 9.88 | | 92 | | 1.25 | | 115 | | 1.57 | | 32 | | 0.44 | |  |
| ***χ^2^*** | 30.633 | |  | | 17.528 | |  | | 31.683 | |  | | 15.641 | |  | | 3.990 | |  | | 21.203 | |  | |  |
| ***P*** | <0.001 | |  | | <0.001 | |  | | <0.001 | |  | | <0.001 | |  | | 0.136 | |  | | <0.001 | |  | |  |
| **FOBT results** |  | |  | |  | |  | |  | |  | |  | |  | |  | |  | |  | |  | |  |
| Non/Negative | 1,691 | | 26.89 | | 1,061 | | 16.86 | | 320 | | 5.09 | | 13 | | 0.21 | | 17 | | 0.28 | | 15 | | 0.23 | |  |
| Positive | 2,056 | | 28.83 | | 1,165 | | 16.33 | | 852 | | 11.95 | | 115 | | 1.61 | | 172 | | 2.41 | | 77 | | 1.08 | |  |
| ***χ^2^*** | 6.301 | |  | | 0.674 | |  | | 197.358 | |  | | 69.954 | |  | | 110.415 | |  | | 34.756 | |  | |  |
| ***P*** | 0.012 | |  | | 0.412 | |  | | <0.001 | |  | | <0.001 | |  | | <0.001 | |  | | <0.001 | |  | |  |
| **Questionnaire results** |  | |  | |  | |  | |  | |  | |  | |  | |  | |  | |  | |  | |  |
| Non/Negative | 2,098 | | 36.41 | | 1,276 | | 22.14 | | 491 | | 8.51 | | 36 | | 0.62 | | 66 | | 1.15 | | 40 | | 0.69 | |  |
| Positive | 1,649 | | 21.53 | | 950 | | 12.40 | | 682 | | 8.91 | | 92 | | 1.20 | | 123 | | 1.60 | | 52 | | 0.68 | |  |
| ***χ^2^*** | 361.818 | |  | | 225.397 | |  | | 0.610 | |  | | 11.566 | |  | | 5.023 | |  | | 0.011 | |  | |  |
| ***P*** | <0.001 | |  | | <0.001 | |  | | 0.434 | |  | | 0.001 | |  | | 0.025 | |  | | 0.916 | |  | |  |
| **Years** |  | |  | |  | |  | |  | |  | |  | |  | |  | |  | |  | |  | |  |
| 2015–2017 | 1,802 | | 25.48 | | 1,060 | | 15.00 | | 694 | | 9.82 | | 94 | | 1.33 | | 122 | | 1.73 | | 24 | | 0.34 | |  |
| 2018–2020 | 758 | | 29.31 | | 401 | | 15.48 | | 230 | | 8.90 | | 20 | | 0.77 | | 42 | | 1.64 | | 22 | | 0.86 | |  |
| 2021–2023 | 1,188 | | 31.54 | | 765 | | 20.31 | | 248 | | 6.60 | | 14 | | 0.37 | | 24 | | 0.65 | | 45 | | 1.20 | |  |
| ***χ^2^*** | 47.822 | |  | | 52.957 | |  | | 32.268 | |  | | 24.962 | |  | | 22.224 | |  | | 28.126 | |  | |  |
| ***P*** | <0.001 | |  | | <0.001 | |  | | <0.001 | |  | | <0.001 | |  | | <0.001 | |  | | <0.001 | |  | |  |
| ***Trend χ^2^*** | 47.193 | |  | | 46.331 | |  | | 31.048 | |  | | 27.830 | |  | | 19.321 | |  | | 27.913 | |  | |  |
| ***P*** | <0.001 | |  | | <0.001 | |  | | <0.001 | |  | | <0.001 | |  | | <0.001 | |  | | <0.001 | |  | |  |
| ***Pearson R*** | 0.059 | |  | | 0.059 | |  | | -0.048 | |  | | -0.043 | |  | | -0.038 | |  | | 0.046 | |  | |  |
| ***P*** | <0.001 | |  | | <0.001 | |  | | <0.001 | |  | | <0.001 | |  | | <0.001 | |  | | <0.001 | |  | |  |
| CRC = colorectal cancer, DR = detection rate, FOBT = fecal occult blood test. | | | | | | | | | | | | | | | | | | | | | | | | |  |

**Supplementary Table 5.** Analysis of CRC staging and follow-up time distribution under different initial screening results

| **Interval time (years)*** | **Initial screening negative** | | | | **Initial screening positive but no colonoscopy** | | | |
| --- | --- | --- | --- | --- | --- | --- | --- | --- |
|  | **Early-stage CRC** | **Mid-/Late-stage CRC** | **Unstaged CRC** | **Total** | **Early-stage CRC** | **Mid-/Late-stage CRC** | **Unstaged CRC** | **Total** |
| ≤1 year | 70 | 119 | 44 | 233 | 151 | 247 | 97 | 495 |
| >1–2 years | 55 | 109 | 60 | 224 | 16 | 33 | 27 | 76 |
| >2–3 years | 53 | 120 | 73 | 246 | 7 | 17 | 14 | 38 |
| >3–4 years | 47 | 134 | 69 | 250 | 9 | 20 | 10 | 39 |
| >4–5 years | 43 | 139 | 72 | 254 | 5 | 16 | 7 | 28 |
| >5–6 years | 51 | 108 | 104 | 263 | 2 | 11 | 11 | 24 |
| >6–7 years | 31 | 96 | 81 | 208 | 1 | 7 | 5 | 13 |
| >7–8 years | 13 | 57 | 56 | 126 | 0 | 2 | 0 | 2 |
| >8–9 years | 3 | 10 | 9 | 22 | 0 | 0 | 0 | 0 |
| **Total** | 366 | 892 | 568 | **1,826** | 191 | 353 | 171 | **715** |
| * The time interval between reporting the initial screening results and reporting the colonoscopy results. | | | | | | |  |  |

CRC = colorectal cancer.
